# Why heel spurs are traction spurs after all

**DOI:** 10.1038/s41598-021-92664-4

**Published:** 2021-06-24

**Authors:** Johann Zwirner, Aqeeda Singh, Francesca Templer, Benjamin Ondruschka, Niels Hammer

**Affiliations:** 1 Department of Anatomy, University of Otago, Dunedin, New Zealand; 2 Institute of Legal Medicine, University Medical Center Hamburg-Eppendorf, Hamburg, Germany; 3 Institute of Legal Medicine, University of Leipzig, Leipzig, Germany; 4 Institute of Macroscopic and Clinical Anatomy, Medical University of Graz, Graz, Austria; 5 Department of Orthopedic and Trauma Surgery, University of Leipzig, Leipzig, Germany; 6 Fraunhofer IWU, Dresden, Germany

**Keywords:** Bone, Cartilage, Tendons

## Abstract

It is unclear whether plantar and posterior heel spurs are truly pathological findings and whether they are stimulated by traction or compression forces. Previous histological investigations focused on either one of the two spur locations, thereby potentially overlooking common features that refer to a uniform developmental mechanism. In this study, 19 feet from 16 cadavers were X-ray scanned to preselect calcanei with either plantar or posterior spurs. Subsequently, seven plantar and posterior spurs were histologically assessed. Five spur-free Achilles tendon and three plantar fascia entheses served as controls. Plantar spurs were located either intra- or supra-fascial whereas all Achilles spurs were intra-fascial. Both spur types consistently presented a trabecular architecture without a particular pattern, fibrocartilage at the tendinous entheses and the orientation of the spur tips was in line with the course of the attached soft tissues. Spurs of both entities revealed tapered areas close to their bases with bulky tips. Achilles and plantar heel spurs seem to be non-pathological calcaneal exostoses, which are likely results of traction forces. Both spur types revealed commonalities such as their trabecular architecture or the tip direction in relation to the attached soft tissues. Morphologically, heel spurs seem poorly adapted to compressive loads.

## Introduction

Heel spurs describe bony outgrows of the calcaneus with plantar^1^ or posterior (“Achilles”)^2^ subtypes, defined by their respective location on the bone. Despite intense research on the topic, it remains unclear to date whether calcaneal spurs present pathological findings or non-pathological exostoses^3^. Plantar calcaneal spurs are more frequent when compared to posterior spurs with a prevalence of 32% and 13%, respectively^4^. Contrary to that, an analysis of calcanei from a prehistoric population revealed a higher incidence of posterior calcaneal spurs when compared to plantar ones with 25 and 3%, respectively^5^. This shows that plantar and posterior spurs are structural features of the foot for a long time of human history but their respective occurrence might be influenced by specific loading patterns. Prehistoric hunters and gatherers, which were presumably unshod likely had different foot biomechanics compared to today’s largely standing overweight and shod population^5^. An increasing incidence of both spur types with age is widely reported^4–7^, presenting evidence that spur development is a physiologically age-related adaption of the calcaneal entheses. It is unknown if plantar or posterior calcaneal spurs develop according to the same basic mechanisms. The hypothesis that calcaneal spurs develop due to soft tissue traction seems to be favoured for posterior spurs, however, this mechanism is increasingly questioned for plantar spurs^2,8–10^. For plantar spurs, it was hypothesised that they develop due to repetitive vertical calcaneus compressions when walking, rather than longitudinal soft tissue-related traction forces^9,10^. Presumably, the compression theory has not been suggested for posterior spurs, as a compressive loading of the Achilles tendon insertion during daily activities such as walking or standing seems unlikely. Wolff’s law^11^, which describes bony remodelling as a result of mechanical loading forms a basic assumption for both the compression and traction theory. According to previous studies that reported the bony architecture of mechano-compromised children in relation to healthy peers, mechanical loading can influence both the trabecular architecture as well as the surface of bones^12^. In summary, trabeculae that are oriented in line with the Achilles tendon and plantar fascia and/or spur growth in the direction of load application of the two soft tissues indicates that the spur results from traction forces. Previous histological studies on the fundamental morphological organization of heel spurs focused on either plantar^13–15^ or posterior^2,16^ spurs, deploying different methodologies. This impairs a direct comparison between these observations. Several authors have suggested considering the Achilles tendon, the calcaneus and the plantar fascia as a functional entity, and including the plantar region in the treatment of Achilles tendon pathologies and vice versa^17–21^. The former is supported by the finding that thick collagen fibres are connecting the Achilles tendon and the plantar fascia via the calcaneus in neonates, forming an apophyseal area, which is ossifying throughout life and leaves only the insertions of the soft tissues into the calcaneus^18^. This given study investigated and compared the histological characteristics of posterior calcaneal spurs, plantar calcaneal spurs, and spur-free controls. The spurs were histologically investigated with regards to trabecular orientation, tip direction in relation to the course of Achilles tendon and plantar fascia collagens and the location of the enthesis in relation to the spur. The following hypothesis was stated: plantar and posterior heel spurs are the result of soft tissue traction from a histological standpoint.

## Material/methods

### Specimen retrieval, X-ray imaging and histology

**Figure 1:**
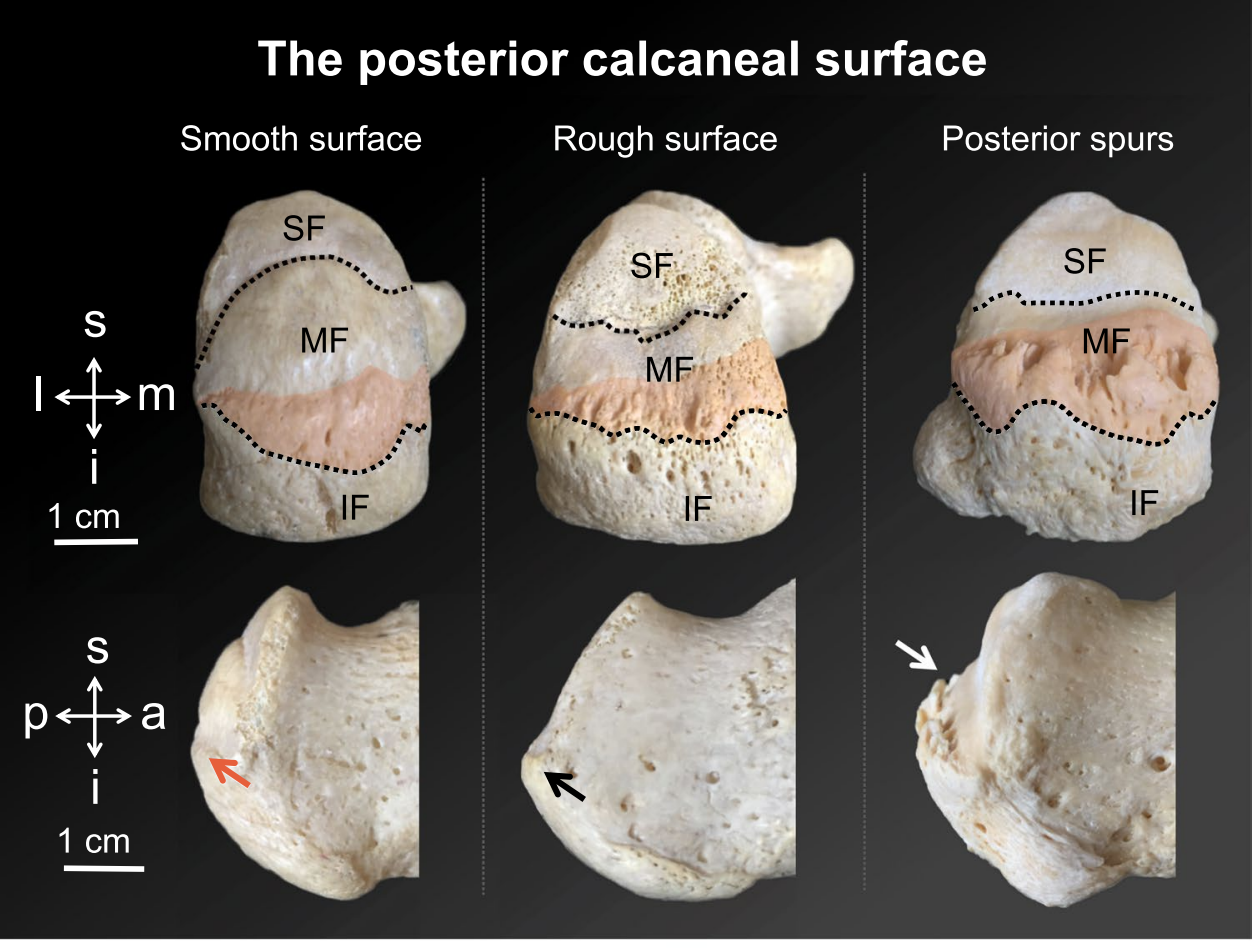
Different shapes of the posterior calcaneal surface are depicted on museum samples of the Department of Anatomy Dunedin, New Zealand. The upper row displays the superior (SF), middle (MF) and inferior facet (IF) of the posterior calcaneal surface in a posterior view. The posteriorly protruding spine separates the MF and the IF (coloured in red). The lower row shows the corresponding lateral view of the samples shown above. Red arrow, barely protruding spine; black arrow, protruding spine; white arrow, protruding spine with posterior spurs; a, anterior; i, inferior; l, lateral; m, medial; p, posterior; s, superior.

**Figure 2:**
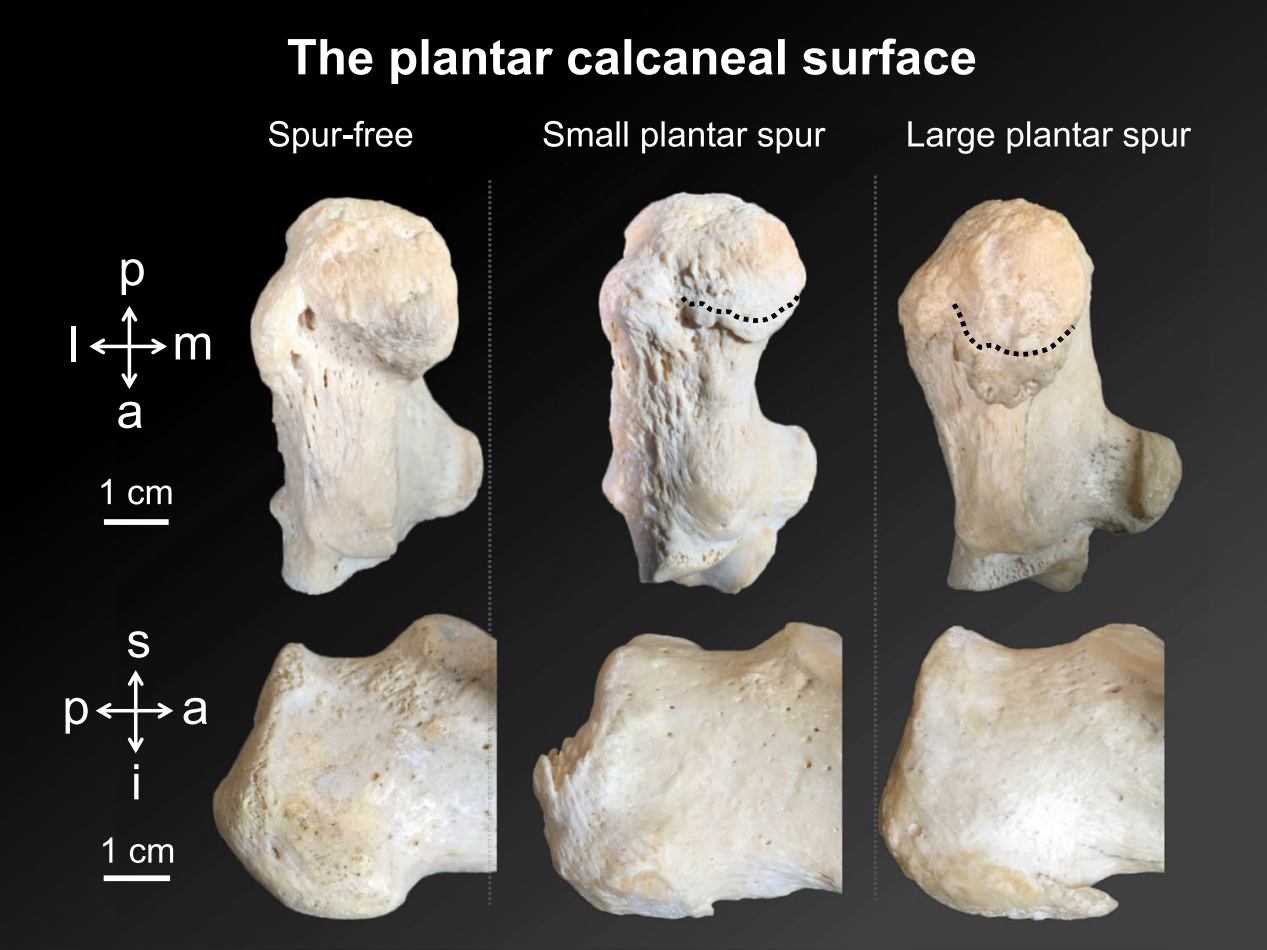
Different shapes of the plantar calcaneal surface are displayed on museum samples of the Department of Anatomy Dunedin, New Zealand. The upper row shows a plantar view of the calcaneus. The dotted lines indicate the base of the spurs. The lower row shows the corresponding medial view to of the samples shown above. a, anterior; i, inferior; l, lateral; m, medial; p, posterior; s, superior.

A total of 19 feet were retrieved from 16 human cadavers (7 ♀, 9 ♂; age at death 79 ± 14, age range 38 to 94 years) at the Department of Anatomy Dunedin, University of Otago, New Zealand. The feet were Crosado-embalmed^22^, which is a combined embalming fluid consisting of ethanol (60%), glycerine (15%), water (15%), phenoxytol (8%), and formalin (2%). Its usability for histological analyses was shown before^22^. The ten left and nine right feet were removed from the lower legs in cadavers approximately ten centimetres proximal to the talocrural joint. They were subsequently imaged using a digital lateral plain X-ray (Carestream Health, Rochester, NY, USA). Based on the X-rays, seven posterior spurs (Fig. 1) were retrieved with the adjacent soft tissues, decalcified in 10% EDTA, dehydrated, paraffin wax-embedded and serially sectioned at 10 μm. Seven plantar spurs (Fig. 2) using the same method. The histology slides were either Giemsa-stained to assess the cartilaginous entheses or silver-stained to evaluate the trabecular architecture and the course of the collagenous soft tissues^23^. Moreover, five Achilles tendon entheses and three plantar fascia entheses with no signs of spur formation were histologically assessed in the same manner serving as control specimens. The cadavers had no known medical history of inflammatory diseases, heel pain or osteoarthritis. None of the cadavers was obese, which was defined as a BMI ≥ 30 kg/m^2^. The University of Otago Ethics Committee approved this study (approval number H17/20), in conjunction with Māori consultation being sought from the Ngai Tāhu Research Consultation Committee. The body donation for research purpose obtained informed consent from the donors or their legal representatives. All methods were carried out in accordance with the relevant guidelines and regulations.

### Data acquisition and analysis

OsiriX software (Pixmeo Sarl, Geneva, Switzerland) was used for the X-ray assessments. Histologically, the location of the plantar spurs was evaluated with regards to the insertion of the plantar fascia into the calcaneus using the silver stain. Similarly, the location of the posterior calcaneal spurs was noted with regards to the location of the Achilles tendon enthesis. Moreover, using the silver staining the presence of trabeculae, the trabecular orientation within all spurs as well as the direction that the tip of the calcaneal spur pointed to (in relation to the predominant collagen orientation of the Achilles tendon or plantar fascia) was evaluated. The presence of fibrocartilage on the outer surface and within the spur of both plantar and posterior calcaneal spurs was assessed using the Giemsa staining. The trabecular orientation was evaluated with the blunt eye under the microscope. Three categories were defined: (A) The trabeculae are strictly oriented along the axis of the Achilles tendon (for posterior spurs) or the plantar fascia (for plantar spurs). (B) The trabeculae are strictly oriented perpendicular to the aforementioned axes of the Achilles tendon or the plantar fascia. (C) The trabeculae lack a preferred orientation.

## Results

### Spur-free calcaneal tuberosity

**Figure 3:**
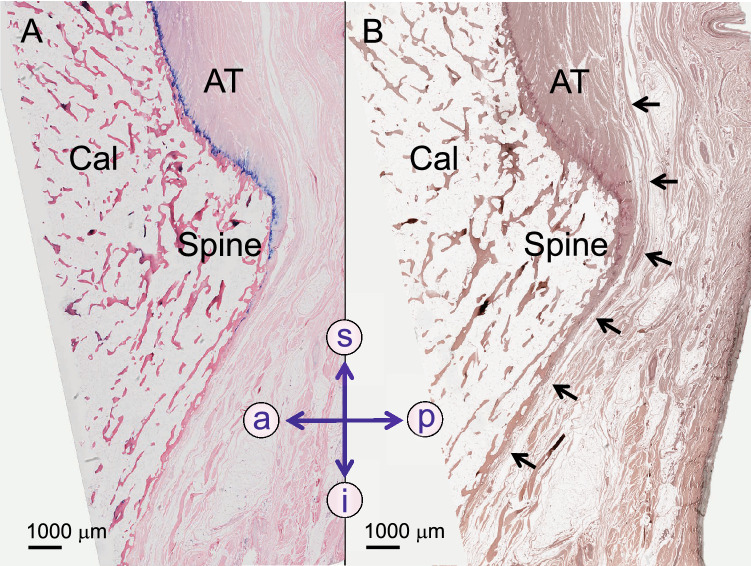
The spur-free protruding spine which separates the middle and inferior facet of the calcaneal tuberosity is shown in a sagittal section. (**A**) The insertion of the Achilles tendon (AT) is shown on a Giemsa-stained slice. Note the blue coloured fibrocartilage of the AT enthesis ending at the level of the spine. (**B**) The slice following the one in A was silver-stained. The black arrows show the paratenon of the AT continuing beyond the spine. a, anterior; Cal, calcaneus; i, inferior; p, posterior; s, superior.

The Achilles tendon inserts into the middle facet of the calcaneal tuberosity that is covered with fibrocartilage (Fig. 3). The paratenon of the Achilles tendon continues its course beyond the protruding spine that separates the middle and inferior facets of the Achilles tendon (Fig. 3).

### Posterior calcaneal spurs

**Figure 4:**
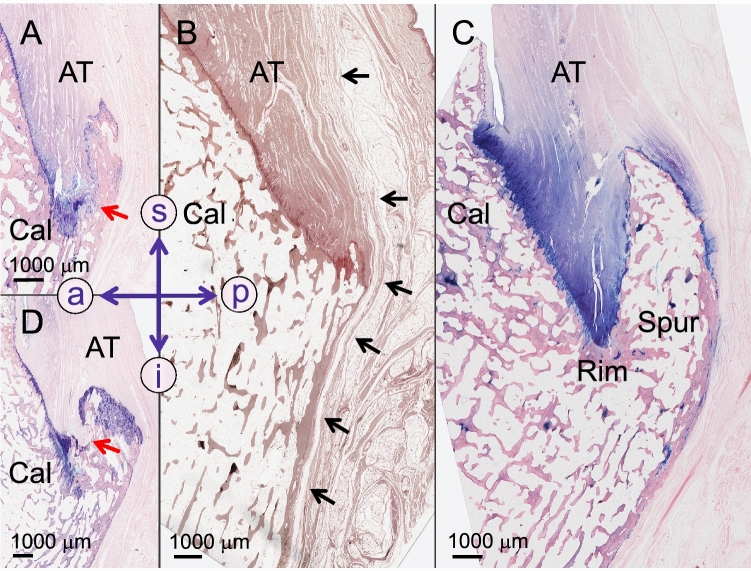
Posterior calcaneal spurs from four different donors are depicted on sagittal slices. (**A**) Giemsa staining. (**B**) The black arrows indicate the paratenon on a silver-stained slice. **C)** Note the blue chondrocytes at the insertion of the Achilles tendon (AT) into the calcaneus on this Giemsa staining. (**D**) Giemsa staining. a, anterior; Cal, Calcaneus; i, inferior; p, posterior; red arrows, tapered areas of the spur; s, superior.

Posterior calcaneal spurs were noted exclusively in the area of the spine that separates the middle and inferior facets of the calcaneal tuberosity. Superficial collagen bundles of the Achilles tendon inserted into the posterior calcaneal spurs, forming a transition zone (Fig. 4). Fibrocartilage surrounded the entire spur in five cases and was limited to its anterior–superior part in two cases. Spur development in the distal area of the Achilles tendon attachment created a rim between the regular surface of the middle facet and the spur in all seven investigated posterior spurs of this study. All but one spur presented an island of fibrocartilage in the rim area. Fibrocartilage islands outside the rim were observed in the proximal posterior area in one spur and distal posterior area in another spur. All spurs presented an intrinsic trabecular orientation, which did not follow a particular pattern. All spur tips pointed in the direction of the preferred collagen orientation of the Achilles tendon. Sporadic chondrocytes were noted in two spurs, fibrocartilage islands within the spur were present in another two spurs. The enthesis of the Achilles tendon did not exceed the base of the spur distally. The paratenon of the Achilles tendon was running past all here investigated spurs (Fig. 4).

### Spur-free medial calcaneal processes

**Figure 5:**
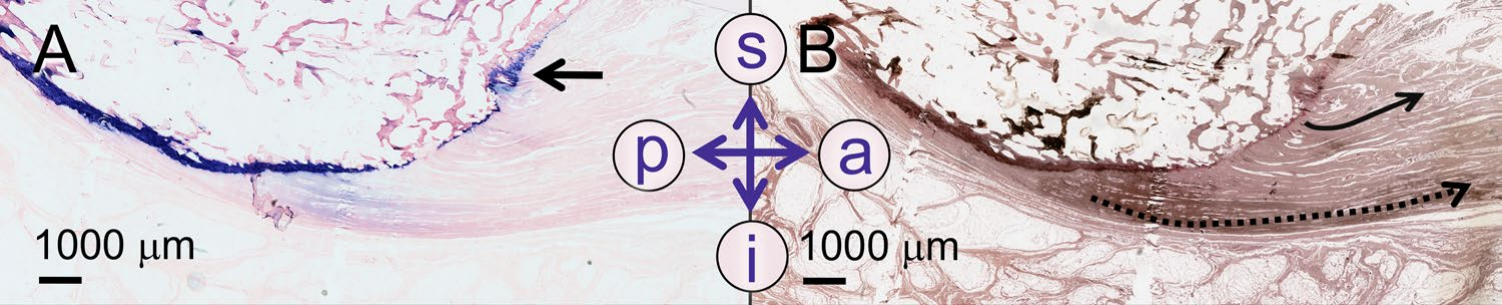
A spur-free medial process of the calcaneus is depicted in a sagittal plane. (**A**) The plantar fascia inserts into the calcaneus. The enthesial cartilage is marked with a black arrow in this Giemsa stain. (**B**) The section in silver staining corresponding to A shows the longitudinal course of plantar fascia collagens, comprising of a superficial layer (SL) originating from the posterior and posterior-inferior aspect of the calcaneus. Plantar fascia collagens of the deeper layer (DL) insert in the anterior-inferior and anterior aspect of the calcaneus, demonstrating a different course of fibres compared to the superficial ones. a, anterior; i, inferior; p, posterior; s, superior.

The plantar fascia inserted into the medial calcaneal process in two distinct layers, a superficial one originating from the posterior and posterior-inferior surface and a deeper layer originating from the anterior-inferior and anterior surface of the medial calcaneal process (Fig. 5). The two layers revealed no strict separation. The superficial layer revealed a longitudinal course of collagens whereas the deeper layers showed a steeper fibre orientation (Fig. 5).

### Plantar calcaneal spurs of the medial calcaneal process

**Figure 6:**
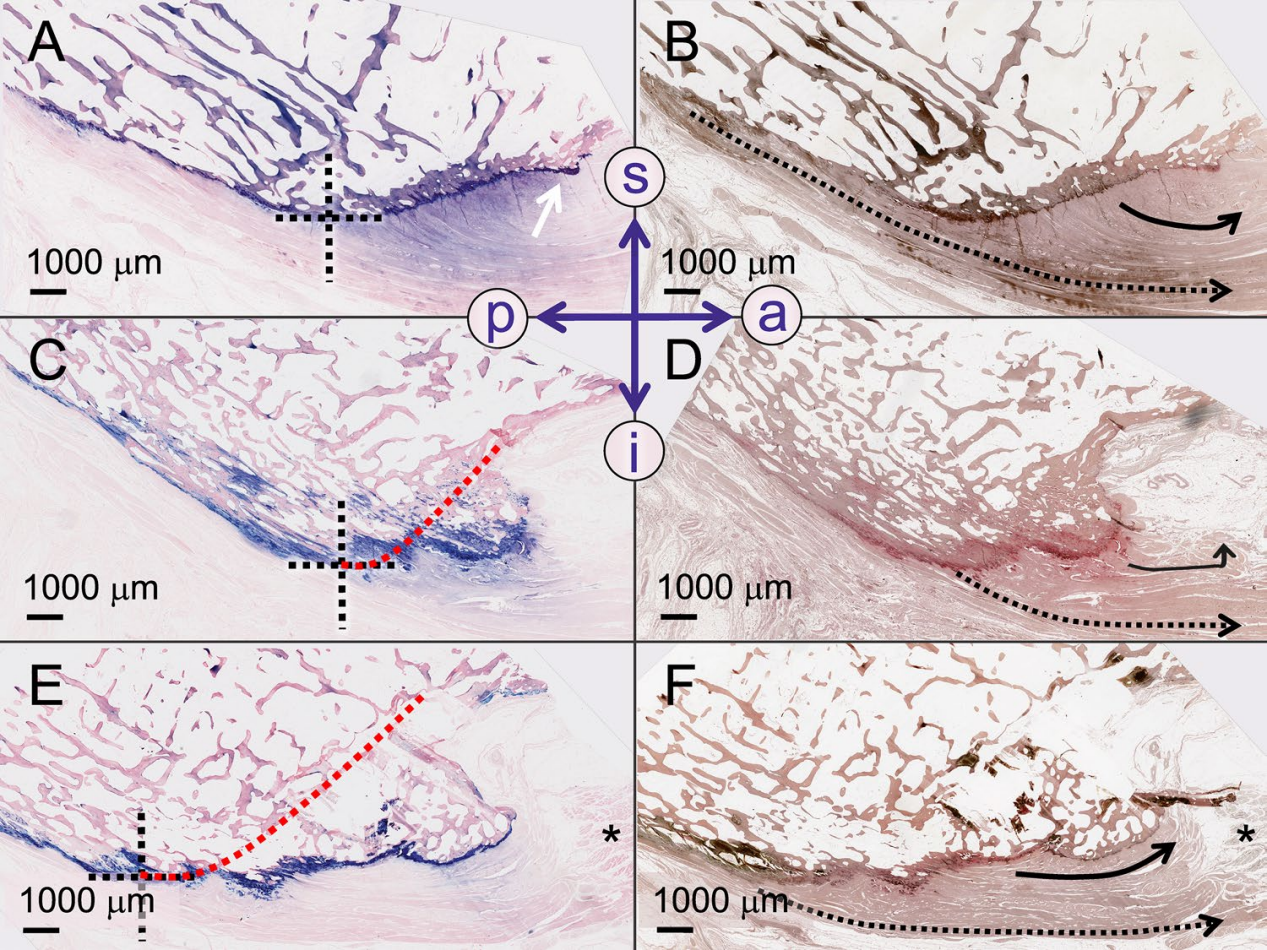
Plantar calcaneal spurs and their pre-stage are depicted from different donors in the sagittal plane. (**A**) A surface irregularity (white arrow), which does represent a developing plantar calcaneal spur is shown (Giemsa stain). The black dotted cross represents a matched dot in (**C**) and (**E**) for comparative reasons. (**B**) Silver staining corresponding to A with superficial (dotted black arrow) and deep (curved black arrow) collagen bundles, (**C**) A spur is present anterior to the estimated border of the calcaneus (red dotted line). (**D**) The silver staining corresponding to (**C**) indicates a different collagen orientation of the deep collagen bundles of the plantar fascia attaching to the spur (black curved arrow) when compared to the superficial collagens directed in a sagittal plane inserting posterior to the spur (black dotted arrow). (**E**) A calcaneal spur is shown with the Giemsa staining and corresponding (**F**) Silver stain. a, anterior; black asterisks (in **E** and **F**), flexor digitorum brevis muscle; curved black arrow, deep collagen bundles; dotted arrow, superficial collagens; p, posterior; i, inferior; s, superior.

**Figure 7:**
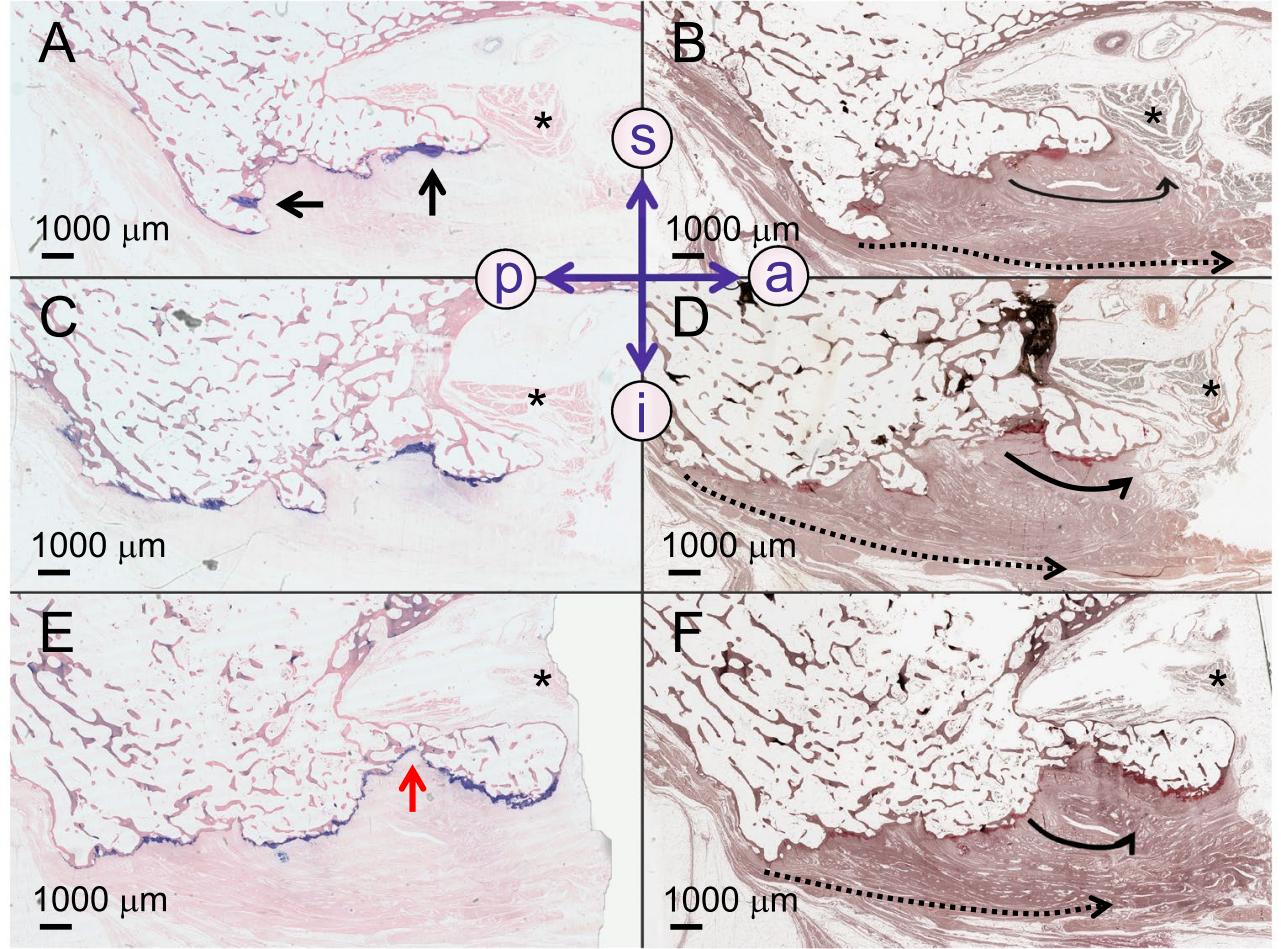
A plantar calcaneal spur of the medial process is shown in a sagittal plane. (**A**) A Giemsa-stained medial slice of a plantar calcaneal spur is depicted with inferior (i) and anterior (a) plantar fascia insertion areas. The black arrow point at enthesial fibrocartilage. (**B**) The silver staining corresponding to (**A**) indicates a different collagen orientation of the plantar fascia attaching to the anterior region of the spur (black arrow) compared to the collagens directed in a sagittal plane inserting the posterior region of the spur (black dotted arrow). (**C**) Giemsa-stained slice located laterally to (**A**), (**D**) Silver-stained slice corresponding to (**C**); curved black arrow, deep collagen bundles; dotted arrow, superficial collagens, (**E**) Giemsa-stained slice located laterally to (**C**); red arrow, tapered area, (**F**) Silver-stained slice corresponding to (**E**); Black asterisks, flexor digitorum brevis muscle; curved black arrow, deep collagen bundles; dotted arrow, superficial collagens; s, superior; p, posterior.

Plantar calcaneal spurs of the deep layer were either located within the fascia (n = 5) and entirely surrounded by an enthesis or superior to the former with an enthesis in their inferior and inferior-anterior areas (n = 2, Fig. 6). Both these spurs of the superficial plantar fascia layer were intra-fascial. Even though at least a thin cartilage layer was observed at the surface of each of the investigated spurs at a 40 × magnification, the entheses appeared to be more prominent in some parts of the spur without following a particular pattern (Fig. 6). All plantar calcaneal spurs contained trabeculae, which lacked a preferred orientation pattern. All tips of the plantar calcaneal spurs pointed in the direction of the predominant collagen orientation of the plantar fascia. Sporadic chondrocytes, as well as fibrocartilage islands, were observed in two of the investigated spurs, respectively. One spur was classified as irregular (Fig. 7), the remaining spurs were deemed simple with a triangular structure that tapered distally.

## Discussion

### Heel spurs likely develop as a result of recurring traction forces

The traction theory assumes that spur formation is a result of soft tissue traction acting on the calcaneus^2^. One proposed mechanism of spur formation involves subperiosteal bleeding that follows a periosteal detachment as a result of soft tissue traction on the enthesis^24^. Alternatively, the soft tissue traction might stimulate the growth of enthesial fibrocartilage, which then forms new bone through enchondral ossification^2,24^. All plantar and posterior calcaneal spurs in this given study contained enthesial fibrocartilage on their surface, supporting the latter hypothesis. This morphologically evidences the close relationship between the observed plantar and posterior spurs and the highly aligned collagenous tissues acting upon them, namely the Achilles tendon and the plantar fascia. The main findings of this study are summarised in Table 1 and highlight the morphological commonalities between plantar and posterior spurs, which can be taken as indicators for a common development mechanism of the two spur types.

**Table 1:** Overview of calcaneal spur characteristics and regular calcaneal features that can be mistaken for true spurs.

	Spur-free AT enthesis	Posterior spur	Spur free PF enthesis	Plantar spur
Spur tip	–	Points towards preferred collagen orientation of AT	–	Points towards preferred collagen orientation of PF
Presence of trabeculae in spurs	–	All spurs	–	All spurs
Trabecular pattern	–	No preferred orientation	–	No preferred orientation
Enthesial fibrocartilage	–	All spurs	–	All spurs
Spur location	–	Intratendinous (in posterior part of AT insertion)	–	Intra- or subfascial
Surface features that can be mistaken for spurs	Calcaneal spine, cortical irregularities (up to 2 mm)*	–	Medial/lateral calcaneal process, cortical irregularities (up to 2 mm)*	

*AT* Achilles tendon, *PF* Plantar fascia.

*Limit adapted from Kirckpatrick et al.^8^ for plantar spurs.

More specifically, based on the absence of a preferred trabecular pattern in this given study that formed the main argument for the compression theory of the development of plantar spurs^9,10^ and the observed morphological commonalities with posterior spurs, it is suggested that both spur types are stimulated by soft tissue traction, which was so far only accepted for posterior spurs^16^. This given study revealed that all posterior spurs were located within the Achilles tendon, whereas plantar spurs were located either within or superior to the plantar fascia. The latter most likely explains why the traction theory is intuitively accepted for posterior spurs but increasingly questioned for plantar ones. In this regard, two aspects should be pointed out: Firstly, all plantar spurs that were located superiorly to the plantar fascia revealed a fibrocartilage enthesis on their inferior and inferior-anterior surface. Therefore, these spurs reveal a close morphological relationship with the plantar fascia, which most likely stimulates their growth caused by traction forces. Secondly, this study highlighted the fact that spurs form complex three-dimensional structures, which were so far most commonly assessed using two-dimensional images both clinically^3,4,7,19,25–29^ and in research^2,10,14,16^. Hence, the enthesis of the plantar fascia in superiorly located spurs can easily be missed, leading to the assumption that the spurs do not develop as a result of external traction forces^9,14^. Alternatively, the traction forces could theoretically also be generated by the flexor digitorum brevis muscle, which is located anteriorly-superiorly adjacent to the plantar fascia spurs^30^. This is, however, opposed by the fact that the fibrocartilage entheses in this study were exclusively orientated towards the plantar fascia. A previous study of our group revealed a complex pattern of collagen bundle organization of the plantar fascia origin with fibres running in a medio-lateral fashion originating from the medial calcaneal process towards the lateral band of the plantar fascia^21^. These collagen bundles appear to be represented by a deep layer of the plantar fascia that was observed to take a different course compared to the superficial layer in this study. Collagen bundles of the superior layers were orientated in a longitudinal fashion running between the medial calcaneal process and the toes. Hence, when the plantar fascia is loaded, the collagen bundles that are attached to the calcaneus pull into different directions and, therefore, can cause complex three-dimensional spur shapes as shown in this study.

### Morphological observations contradict spur formation due to compressive loading

The compression theory of calcaneal spur formation suggests that plantar spurs develop as a consequence of repetitive compressive loading through ground reaction forces causing microtrauma of the enthesis region^9,14^. It was suggested that the latter induces a periosteal reaction, inducing bone growth^14^. The compressive theory was derived from the observation of vertically aligned trabeculae within the spur^9,14^. It was assumed that the vertical orientation of the trabeculae develops as a result of a vertically orientated ground reaction force vector following Wolff’s law^14^. This given study using a critical qualitative assessment of the histological slides revealed that the trabeculae in both plantar and posterior spurs followed a complex pattern with no preferred or predictable orientation. In fact, the trabecular orientation was so far only assessed upon gross examination^9,14^ and quantitative analysis using a large sample size is missing to date. Moreover, previous research that favours the compression theory already observed that a vertical trabecular orientation is absent in some plantar spurs^9^. In summary, we conclude that solely based on the trabecular orientation within the spur there is no convincing evidence supporting neither the traction nor the compression theory. The strongest morphological evidence supporting the traction theory in this given study results from the fact that all spurs presented an enthesis of the Achilles tendon or the plantar fascia and additionally pointed into the direction of these tissues. Following the observation that all spurs contained plantar fascia entheses, it can be assumed that the plantar fascia considerably loads the spur whenever it is strained, e.g., during walking or standing. We suggest that the trabecular orientation of both plantar and posterior spurs is determined by the resultant force acting on the bone. This involves both ground reaction and pulling forces. This study revealed that both plantar and posterior spurs have a complex three-dimensional architecture such as tapered areas in the proximal spur part close to its base or spurs that taper towards their plantar side. Biomechanically, a tapered area close to the spur base with a bulky tip implies that some spurs are weaker closer to the base than in the distal tip area when compressively loaded. Moreover, the plantar tapering of some spurs contradicts the general anatomical observation that bones are wider in their weight-bearing surfaces, which is true for the epiphyses of all long bones. From our perspective, this observation opposes the idea that the spur forms as an adaption to compressive mechanical loads or the spur is considerably loaded compressively at all. In line with this theory, calcaneal spurs were noted as early as in a 9-month-old infant with a severe metatarsus varus deformity^27^. We suspect that spur formation in the former case was induced by tendinous pulling forces rather than compressive loads as infants will not extensively load their calcanei at this age. Posteriorly directed plantar spurs of the calcaneus were also noted incidentally in a 7-year-old boy with flexion deformities of the toes as well as a 3-year-old boy with a suspected fracture^27^.

All plantar and posterior spurs that were investigated in this given study presented a trabecular architecture indicating that the spur formation itself is very likely a non-pathological adaptation process to the forces acting on the superficial calcaneus instead of a pathological proliferative bone formation. However, after their adaptive formation, spurs might be overloaded easily given their location on the posterior and inferior calcaneus leading to secondary inflammatory changes, which can be seen in histology. Based on the aforementioned observations, it can be suggested that both plantar and posterior calcaneal spurs are traction spurs. Thus, the stated hypothesis can be accepted. These are most likely the results of increased traction forces or an altered force transmission on the calcaneal entheses that are related to age^4–7^ and body weight^10,31–33^. It is widely accepted that spur incidence increases with increasing age^4–7^. The biomechanical properties of human ligaments tend to show site-dependent changes in tissue elasticity with age^34,35^. While several studies investigated the relationship between age and elasticity of the human Achilles tendon with contradictory results^36,37^, the plantar fascia has just recently been mapped for such investigations^21^. Altered elastic properties of the Achilles tendon and plantar fascia will impact on their potential to store and release elastic potential energy^38^. Altered tendon biomechanics, irrespective of whether the tendon is too overly or soft, might lead to overloads on the tendinous entheses, which cause the development of spurs according to the abovementioned mechanisms described by Bergmann^24^ and Rufai^2^.

An age-related elasticity change of the plantar fascia and the Achilles tendon might alter their energy storage potential during walking or standing, which might stimulate the development of traction spurs on their calcaneal entheses. Similarly, this mechanism may apply to patients suffering from plantar fasciitis. Plantar fasciitis was shown to predominantly represent degeneration of the plantar fascia rather than an inflammation^39^. Higher spur incidences were observed in females younger than 50 years compared to males^7^. Frequent walking in high-heeled shoes was the suspected reason for this observed difference, which fits well with the favoured traction theory of this study. Dorsally extended toes in high-heeled shoes strengthen the plantar fascia and, therefore, likely produce traction forces at its calcaneal enthesis. The increased spur incidence of patients suffering from osteoarthritis can be attributed to an altered force-transmission on the calcaneal entheses due to a pathological gait pattern^40^. However, it should be emphasised that the evidence for an increased incidence of calcaneal spurs in patients with osteoarthritis is weak^10^ and some studies in this regard appear to be biased by older osteoarthritis cohorts compared to (younger) controls^26,29^. The incidence of calcaneal spurs has been shown to significantly increase with body weight^10,31–33^, which likely increases the applied loads to the longitudinal arch and, thus, the loads applied to the plantar fascia and its respective calcaneal enthesis. In line with Benjamin et al.^16^ it can be stated that increased pulling forces on the calcaneal entheses lead to the spur formation to increase the calcaneal attachment area of both the plantar fascia and the Achilles tendon. Regarding the proven correlation between body weight and spur incidence^10,31–33^, overweight should be avoided to limit spur formation in the first place. If spurs are truly stimulated by traction forces as concluded in this given study, higher weights likely cause increased traction forces on the calcaneal entheses, which is well conceivable as higher body weights lead to increased stresses on the plantar arches of the foot. However, a normal trabecular architecture within the spur indicates that spur formation is an adaptation of the bone to its biomechanical needs as outlined above. Hence, the main clinical implication might rather be to avoid overloading of the spurs due to their poor adaptation to compressive loading forces as indicated by tapered areas that represent notches as these form preferential failure points when loaded compressively. Heel spurs seem to be a non-pathological adaptation of a well-balanced load-transmission between the calcaneus and its surrounding soft tissues, which explains the comparatively high incidences in randomly chosen cohorts^4–7^. However, compressive overstimulation during daily activities might cause an inflammation of these exophytes, which led to the image that calcaneal spur formation is a pathological and painful process in the first place^14^.

## Limitations

This study is limited in sample size due to the fixed number of cadaveric specimens that were allocated to this project. Therefore, it has to be considered that the stated results might be biased by the limited sample size. The stated results might not apply to age cohorts that were not included in the age range of this study. It cannot be excluded that the embalming fluid and histological processing influenced the staining outcome of the histological sections or the here made radiographic observations.

## Conclusions

Plantar and posterior heel spurs constitute non-pathological exostoses of the calcaneus. Histological analysis of both spur types revealed commonalities such as their trabecular architecture or the tip direction in relation to the attached soft tissues. Based on the morphological analyses it is suggested that both plantar and posterior spurs are the result of tissue traction. From a morpho-mechanical perspective, calcaneal spurs seem poorly adapted to compressive loads.
